# Methylprednisolone in the treatment of lymphangioleiomyomatosis

**DOI:** 10.1186/s43168-022-00127-9

**Published:** 2022-05-26

**Authors:** Ventour Dale, Geosanne Thannoo, Shammi Ramlakhan

**Affiliations:** 1 The University of the West Indies, Saint Augustine, Trinidad and Tobago; 2 Eric Williams Medical Sciences Complex, Champs Fleurs, Trinidad and Tobago; 3 Sheffield Children Teaching Hospital NHS Trust, Sheffield, UK

## Abstract

This case report proposes the use of pulsed methylprednisolone in a patient with lymphangioleiomyomatosis (LAM) who was invasively ventilated. A 38-year-old, East Indian gravida 4, para 1^+2^, patient with worsening respiratory function with spontaneous pneumothoraxes and hypoxemia was pulsed with methylprednisolone leading to rapid resolution of respiratory failure.

The author proposes pulsed methylprednisolone in ventilated LAM patients, which gives another option to patients which resulted in alleviation of hypoxemia and maintaining foetal viability.

## Introduction

This manuscript proposes the use of pulsed methylprednisolone in a patient with lymphangioleiomyomatosis (LAM) who was invasively ventilated.

**Fig. 1:**
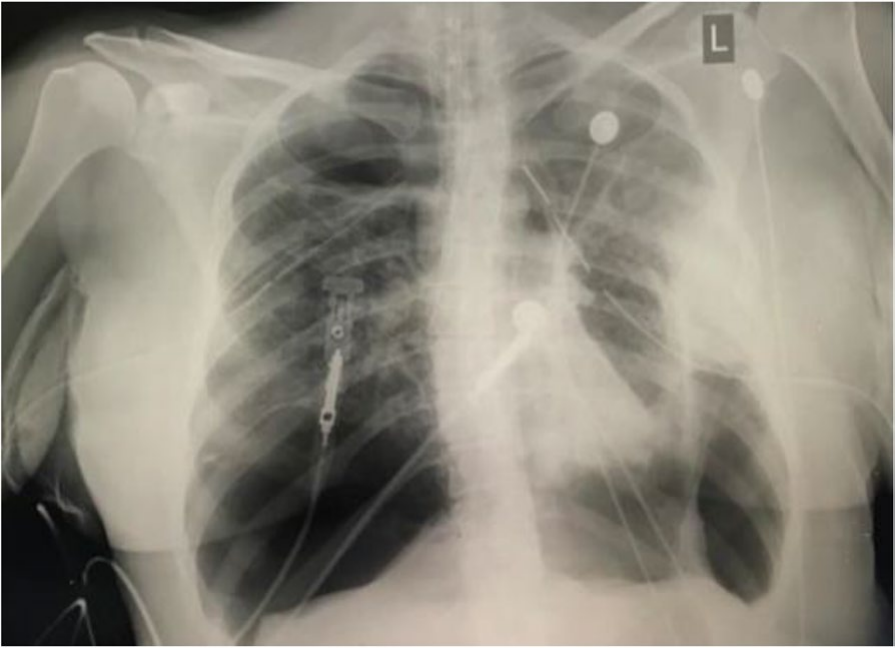
Eric Williams Medical Sciences Complex for thoracic surgical management of bilateral spontaneous pneumothoraxes and a left-sided hydrothorax

RR, an East Indian 38-year-old female, non-smoker, G4P1^+2^ and currently thirteen (13) weeks pregnant, was referred to Eric Williams Medical Sciences Complex for thoracic surgical management of bilateral spontaneous pneumothoraxes and a left-sided hydrothorax (see Fig. 1). The patient originally complained of a 5-day history of shortness of breath. Her Covid-19 PCR was negative, and a CTPA revealed no evidence of pulmonary embolism, but there was a left-sided pneumothorax and effusion suggestive of haemothorax with associated atelectasis. There were also numerous thin-walled cysts seen scattered throughout both lungs.

The differential diagnoses included lymphangioleiomyomatosis (LAM), pulmonary Langerhans cell histiocytosis (LCH) or emphysema. No mediastinal or hilar adenopathy was seen.

### Clinical findings

When reviewed by critical care, the patient had two (2) chest drains in situ, and a chylothorax on the left side was drained. She was scheduled for a left-sided video-assisted thoracoscopic surgery, but 3 days after, her admission was found unresponsive and hypoxic.

Her arterial blood gas at the time revealed type 2 respiratory failure with a P_a_CO_2_ 98 mmHg, pulse 135 bpm and BP 145/96 mmHg, and on examination, she had decreased air entry in the right lung. A right 14 G needle was inserted in the second intercostal space, mid-clavicular line, and the patient was then intubated. There was a gush of air but no improvement in oxygenation or right air entry.

Her post-intubation oxygenation did improve with her saturations increasing to 98% with bag and mask ventilation, but her chest X-ray revealed a persistent right pneumothorax. A third thoracostomy tube was inserted with sustained improvement in her oxygenation.

The working diagnosis was LAM at this point, and an obstetric opinion was obtained. The patient was admitted to the intensive care unit, and the foetal age confirmed as 26/40 with a foetal heart beat present. Dexamethasone was initiated for foetal lung maturity and ventilation, and sedation and basic ICU support continued.

The type 2 respiratory failure started improving with ventilation on volume-controlled ventilation initial settings: FiO2 0.6 PEEP 5 rate 20 VT 400. Dexamethasone 4 mg was initiated, and her care in ICU involved lung-protective ventilation and supportive care.

Her Glasgow Coma Score (GCS) is always 11T/15 when off sedation. The patient required a small dose of noradrenaline that was weaned, and ventilation was challenging — still presenting with type 2 respiratory failure, and lung-protective ventilation was instituted.

**Fig. 2:**
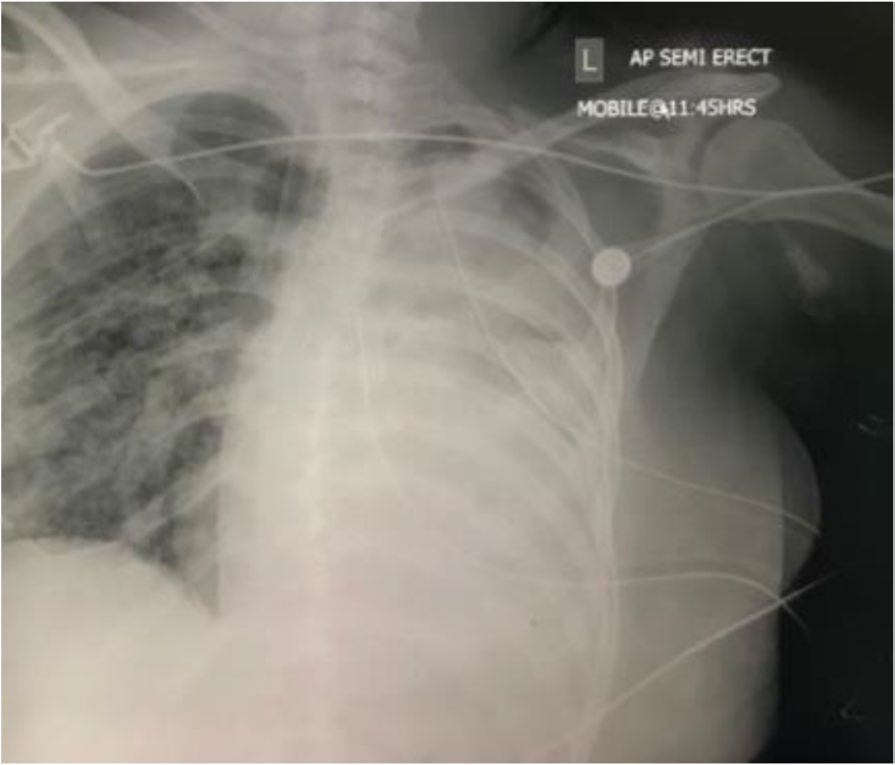
Left main bronchus completely occluded with clots which were removed

**Fig. 3:**
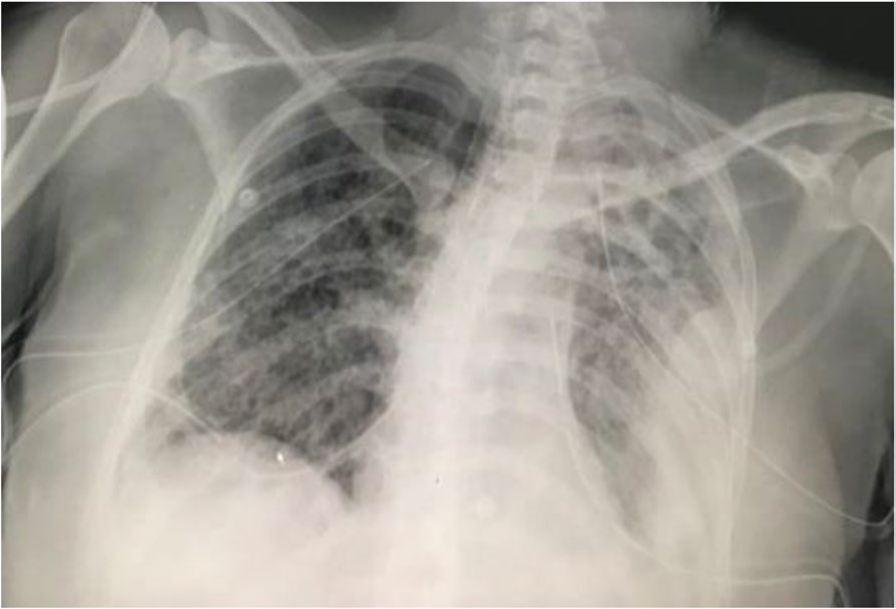
Diagnostic/investigations

After worsening lung condition and a CXR that showed total opacity of left lung on 20/02/2021, a bronchoscopy was scheduled to be done in ICU. Bronchoscopy showed bloody secretions in trachea and right main bronchus; left main bronchus completely occluded with clots which were removed (Figs. 2 and 3).

### Diagnostic/investigations

**Fig. 4:**
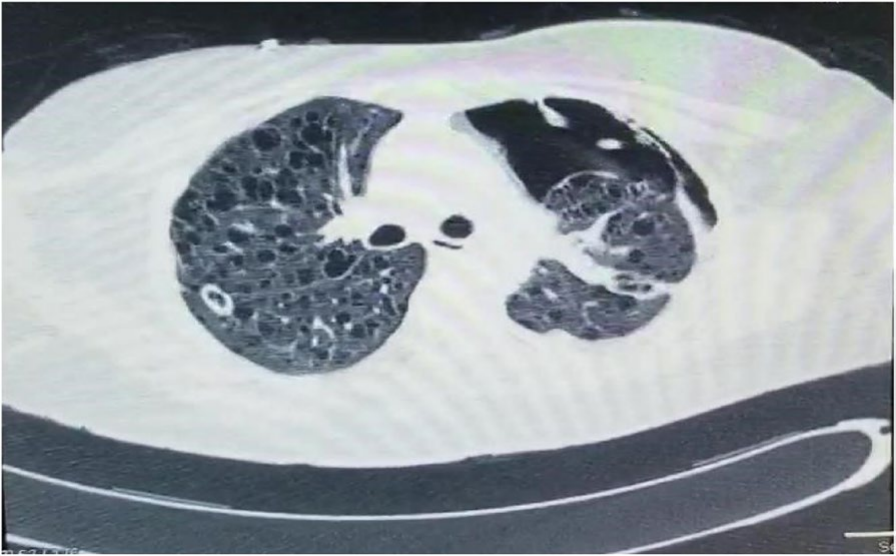
Another bronchoscopy was done due to noted increased 133 peak pressures

The patient was also being treated with vancomycin and meropenem for a possible nosocomial pneumonia, and on 22/2/2021, a CT brain and chest (Fig. 4) was done as follows:

- Showed nil acute intracranial findings
- Tiny right pleural effusion
- Small bilateral pneumothoraxes
- Small left pneumothorax
- Innumerate small regular lung cysts diffusely distributed throughout both lung fields with background ground glass opacification of lung parenchyma consider LAM.

On 26/2/21, another bronchoscopy was done due to noted increased peak pressures —no abnormal finding; her airways were clear.

Other investigations included the following:

- HIV ELISA was negative; Covid-19 PCR was also negative.
- Bronchoalveolar lavage (BAL) (22/2/21) culture detected pseudomonas and Enterobacteriaceae BAL (22/2/21); there was no fungal growth.
- Blood culture (14/2/21) detected pseudomonas sensitive to piperacillin-tazobactam but resistant to vancomycin and meropenem. Her urine on (13/2/21) revealed nil bacterial growth.

### Intervention

The patient completed 3 days of antibiotics, but her ventilatory support remained very high; she was then pulsed on methylprednisolone 1 g intravenously for 3/7 and then weaned to prednisolone 60 mg daily. Her ventilatory settings reduced over 48 h, but she remained confused, weak and agitated. A tracheostomy was performed on 28/2/21, the patient was able to be weaned onto CPAP and transferred to the High Dependency Unit.

### Outcome and follow-up

The patient was weaned to room air, but due to the risk of recurrence and worsening of lung function, it was decided at a multidiscipline team meeting with obstetrics, thoracic surgical, and medical that a medical abortion was the next step in her management to reduce her risk of recurrence with such lung parenchymal damage. She was induced 2 days later after consent by both mother and partner; she tolerated delivery of the foetus which did not survive.

RR continued to improved, she was placed on 5 mg prednisolone maintenance, the tracheostomy was decannulated a week later and the patient was discharged to the home.

## Discussion

The classic clinical presentation of LAM is quite distinctive; women of childbearing age present with spontaneous pneumothorax, chylothorax, haemoptysis and slowly progressive dyspnoea [1]. Microscopically, the lungs are characterised by cystic air spaces and a nodular proliferation of abnormal smooth muscle cells (LAM cells). LAM cells accumulate in the alveolar walls, collagen and elastic fibres are partially degraded, and oedema, haemorrhage and haemosiderin-laden macrophages are seen around the alveoli [2].

Not all patients with LAM require tissue biopsy for a definitive diagnosis as the disease has a characteristic of CT appearance in the majority of cases. CT scanning shows multiple thin-walled cysts scattered throughout the lung fields in an even distribution with normal intervening lung parenchyma. Therefore, patients with suspected LAM should have a high-resolution CT of the thorax and a CT of the abdomen to examine for the presence of angiomyolipoma and other lymphatic involvement. Where the CT scan is not characteristic of LAM, a lung biopsy should be obtained for a definitive diagnosis [3].

The diagnosis of Langerhans cell histiocytosis was not entertained based on the total lung involvement, no association with smoking and the CT appearance [4].

The treatment usually involves sirolimus, antihormonal therapy including oophorectomies and progestogens and lung transplantation. All these modalities have serious detrimental potential effects on the foetus, and we initiated pulsed methylprednisolone therapy which rapidly stabilized the clinical lung deterioration, and we were able to wean the ventilatory support very rapidly, within 2 days, and decrease the patient’s respiratory support.

A medical abortion was carried out in this case to decrease her risk of pneumothorax recurrence and further lung deterioration.

## Conclusion

The author proposes that pulse methylprednisolone needs to be the first-line treatment in patient with and suspected to having LAM as it decreases the progression of the disease and also reduces the length of time on the ventilator.

## Data Availability

A file was uploaded to the supplementary data. Also, patient’s note is available to access.
